# Residential Structural Racism and Prevalence of Chronic Health Conditions

**DOI:** 10.1001/jamanetworkopen.2023.48914

**Published:** 2023-12-21

**Authors:** Dinushika Mohottige, Clemontina A. Davenport, Nrupen Bhavsar, Tyler Schappe, Michelle J. Lyn, Pamela Maxson, Fred Johnson, Arrianna M. Planey, Lisa M. McElroy, Virginia Wang, Ashley N. Cabacungan, Patti Ephraim, Paul Lantos, Sarah Peskoe, Joseph Lunyera, Keisha Bentley-Edwards, Clarissa J. Diamantidis, Brian Reich, L. Ebony Boulware

**Affiliations:** 1 Institute for Health Equity Research, Department of Population Health, Icahn School of Medicine at Mount Sinai, New York, New York; 2 Barbara T. Murphy Division of Nephrology, Department of Medicine, Icahn School of Medicine at Mount Sinai, New York, New York; 3 Department of Biostatistics and Bioinformatics, Duke University, Durham, North Carolina; 4 Center for Community and Population Health Improvement, Duke Clinical and Translational Science Institute, Duke University, Durham, North Carolina; 5 Department of Family Medicine and Community Health, Duke University, Durham, North Carolina; 6 Department of Health Policy and Management, Gillings School of Global Public Health, Chapel Hill, North Carolina; 7 Cecil G. Sheps Center for Health Services Research, University of North Carolina at Chapel Hill, Chapel Hill; 8 Duke Global Health Institute, Duke University, Durham, North Carolina; 9 Department of Pediatrics, Duke University, Durham, North Carolina; 10 Duke Cancer Institute, Duke University, Durham, North Carolina; 11 Division of General Internal Medicine, Department of Medicine, Duke University, Durham, North Carolina; 12 Division of Nephrology, Department of Medicine, Duke University, Durham, North Carolina; 13 Division of Abdominal Transplant Surgery, Department of Surgery, Duke University, Durham, North Carolina; 14 Department of Population Health, Duke University, Durham, North Carolina; 15 Samuel DuBois Cook Center on Social Equity, Duke University, Durham, North Carolina; 16 Feinstein Institutes for Medical Research, Northwell Health, Manhasset, New York; 17 Department of Statistics, North Carolina State University, Raleigh; 18 Wake Forest University School of Medicine, Winston Salem, North Carolina

## Abstract

**Importance:**

Studies elucidating determinants of residential neighborhood–level health inequities are needed.

**Objective:**

To quantify associations of structural racism indicators with neighborhood prevalence of chronic kidney disease (CKD), diabetes, and hypertension.

**Design, Setting, and Participants:**

This cross-sectional study used public data (2012-2018) and deidentified electronic health records (2017-2018) to describe the burden of structural racism and the prevalence of CKD, diabetes, and hypertension in 150 residential neighborhoods in Durham County, North Carolina, from US census block groups and quantified their associations using bayesian models accounting for spatial correlations and residents’ age. Data were analyzed from January 2021 to May 2023.

**Exposures:**

Global (neighborhood percentage of White residents, economic-racial segregation, and area deprivation) and discrete (neighborhood child care centers, bus stops, tree cover, reported violent crime, impervious areas, evictions, election participation, income, poverty, education, unemployment, health insurance coverage, and police shootings) indicators of structural racism.

**Main Outcomes and Measures:**

Outcomes of interest were neighborhood prevalence of CKD, diabetes, and hypertension.

**Results:**

A total of 150 neighborhoods with a median (IQR) of 1708 (1109-2489) residents; median (IQR) of 2% (0%-6%) Asian residents, 30% (16%-56%) Black residents, 10% (4%-20%) Hispanic or Latino residents, 0% (0%-1%) Indigenous residents, and 44% (18%-70%) White residents; and median (IQR) residential income of $54 531 ($37 729.25-$78 895.25) were included in analyses. In models evaluating global indicators, greater burden of structural racism was associated with greater prevalence of CKD, diabetes, and hypertension (eg, per 1-SD decrease in neighborhood White population percentage: CKD prevalence ratio [PR], 1.27; 95% highest density interval [HDI], 1.18-1.35; diabetes PR, 1.43; 95% HDI, 1.37-1.52; hypertension PR, 1.19; 95% HDI, 1.14-1.25). Similarly in models evaluating discrete indicators, greater burden of structural racism was associated with greater neighborhood prevalence of CKD, diabetes, and hypertension (eg, per 1-SD increase in reported violent crime: CKD PR, 1.15; 95% HDI, 1.07-1.23; diabetes PR, 1.20; 95% HDI, 1.13-1.28; hypertension PR, 1.08; 95% HDI, 1.02-1.14).

**Conclusions and Relevance:**

This cross-sectional study found several global and discrete structural racism indicators associated with increased prevalence of health conditions in residential neighborhoods. Although inferences from this cross-sectional and ecological study warrant caution, they may help guide the development of future community health interventions.

## Key Points

**Question:**

Are indicators of residential neighborhood (census block group) structural racism associated with the prevalence of chronic health conditions?

**Findings:**

In this cross-sectional study of 150 neighborhoods in Durham County, North Carolina, an increased burden of structural racism indicators, including global (lower percentage of White individuals, lower economic and racial spatial advantage, and higher area deprivation) and discrete (higher reported violent crimes, evictions, poverty, unemployment, uninsurance, and child care center density, and lower election participation, income, and education) measures were associated with greater neighborhood prevalence of chronic kidney disease, diabetes, and hypertension.

**Meaning:**

These findings suggest that structural racism indicators should be considered when developing interventions to improve neighborhood health.

## Teaser

This cross-sectional study examines associations of structural racism indicators with neighborhood prevalence of chronic kidney disease, diabetes, and hypertension in 150 neighborhoods in Durham County, North Carolina.

## Introduction

Racially and ethnically minoritized individuals, ie, individuals who experience minoritization due to historic and present-day sociopolitical power differentials (eg, American Indian, Black, and Hispanic or Latino individuals) experience substantially greater prevalence of chronic conditions including chronic kidney disease (CKD), diabetes, and hypertension compared with nonminoritized White individuals.^1,2,3^ Epidemiological analyses illustrate clustering of poor health outcomes and structural disadvantage at the regional, county, and residential neighborhood levels.^4,5,6,7,8,9,10,11,12^ For instance, neighborhood-level characteristics, including racialized structural disadvantages^13^ (eg, poverty), disproportionately burden racially and ethnically minoritized communities and have been associated with greater COVID-19 incidence and morbidity.^14,15^ These findings highlight the need to understand the contribution of macrocosmic neighborhood-level forces, including conditions resulting from structurally racist policies, to health inequities.^16^

The term *structural racism* signifies how “societies foster racial discrimination through mutually reinforcing systems of housing, education, employment, earnings,…health care, and criminal justice. These patterns and practices in turn reinforce discriminatory beliefs, values, and distribution of resources.”^17^ A well-known historical example of structurally racist US policies is 1930s federally sanctioned housing loan policies (termed *redlining*), which systematically restricted equitable housing access, resulted in limited access to health-enabling resources, and increased health-harming exposures among Black and other racially and ethnically minoritized individuals over generations.^8,18,19^ These policies and others have resulted in concentrated disadvantage and disinvestment in many neighborhoods, producing significant neighborhood-level social inequity across the US.^5,12,13,17,20,21,22^

Efforts to elucidate potential targets for interventions designed to mitigate residential neighborhood health inequities are nascent.^23^ We examined the association of global and discrete indicators of structural racism with residential neighborhood prevalence of CKD, hypertension, and diabetes––chronic health conditions for which racial and ethnic disparities have long been described.

## Methods

This cross-sectional study was approved by the Duke University institutional review board with a waiver of consent, because electronic health record (EHR) data were deidentified and presented in aggregate for each census block group. This study is reported following the Strengthening the Reporting of Observational Studies in Epidemiology (STROBE) reporting guideline.

### Study Design

We conducted a cross-sectional ecological study to assess associations of neighborhood structural racism indicators with prevalence of chronic conditions. We conducted analyses using data collected in 2017 to 2018. Residential neighborhoods included 150 census block groups in Durham County, North Carolina, a midsize metropolitan county in the southeastern US, for which residents’ deidentified electronic health records and data on structural racism indicators were publicly available. Electronic health record data were only available for Durham County, limiting our analyses to this geographic region.

### Setting and Study Neighborhoods

We defined residential neighborhoods as geographic census block groups, which represent the smallest statistical subdivision for which data are released and typically contain 600 to 3000 individuals.^24^ We defined race and ethnic categories using US Census categories from the 2010 Decennial US Census, including Asian, Black or African American (hereafter, *Black*), Hispanic or Latino, Indigenous (ie, American Indian and Alaska Native), and White.^25,26^ Durham County (population 269 702 in 2019) is racially and ethnically diverse, with US Census data indicating 38% of residents identified as Black, 14% of residents identified as Hispanic or Latino, and 42% of residents identified as White. Durham County is undergoing rapid gentrification and has significant disparities in median household income and education.^27,28,29,30^

### Prevalence of CKD, Diabetes, and Hypertension

We quantified the prevalence of CKD, diabetes, and hypertension in residential neighborhoods separately. We did not consider additional conditions. We derived aggregate estimates of CKD, diabetes, and hypertension prevalence among adults aged 18 years and older in each residential neighborhood using data from the Durham Neighborhood Compass,^25,28^ which publishes data derived from deidentified electronic health records of individuals residing in Durham County and receiving care at 2 local health care systems, Duke University Health System, a private not-for-profit health system, and Lincoln Community Health Center, a federally qualified health center. These data represent more than 85% of Durham County residents.^28,31^ The presence of each chronic condition was determined based on laboratory values and diagnosis codes from the calendar year specified (eMethods in Supplement 1).^22^ CKD was ascertained based on 2017 laboratory values and defined using 2012 Kidney Disease Improving Global Outcomes criteria of stage 3 and above,^32^ diabetes was ascertained based on 2017 values and defined as individuals who had type 2 diabetes according to *International Classification of Diseases, Ninth Revision (ICD-9)* and *International Statistical Classification of Diseases and Related Health Problems, Tenth Revision (ICD-10)* codes or based on a hemoglobin A_1c_ measurement of 6.5% or above (to convert to proportion of total hemoglobin, multiply by 0.01),^33^ and hypertension was assessed based on 2018 values and defined as individuals who had a systolic blood pressure greater than 140 mm Hg or diastolic blood pressure greater than 90 mm Hg within a 12-month period, excluding inpatient readings (eMethods in Supplement 1).^34,35,36^ Condition prevalence data were masked if there were fewer than 3 individuals in a block group.

### Sample Size, Exclusions, and Data Sources

We did not have a priori hypotheses regarding effect sizes we might observe and therefore did not conduct power calculations. We excluded block groups that were not residential or that lacked data. To characterize structural racism, we used variables from multiple data sources (eAppendix 1 in Supplement 1), including local data contained in the Durham Neighborhood Compass (eg, Durham Police, Housing Authority)^25,28^ and national data from the American Community Survey (ACS),^28^ the Eviction lab,^37^ and Mapping Police Violence.^38^

### Global Structural Racism Indicator Variable Definitions

Although no single definitive measure of structural racism exists,^39,40,41,42^ racialized residential and economic segregation have been outlined as fundamental mechanisms through which structurally racist policies have created spatial concentrations of disadvantage in racially and ethnically minoritized populations and relative advantage in White populations (eg, redlining, housing and lending policies).^17,20,21,22,43,44,45,46,47^ We characterized global indicators of structural racism as those broadly reflecting racialized and economic neighborhood segregation among neighborhoods (eAppendix 1 and eAppendix 2 in Supplement 1). These indicators serve as markers of privilege, spatial segregation, and deprivation formed through structurally racist policies.

#### Neighborhood Percentage of White Residents

We used percentage of neighborhood residents identifying as White as a global measure because structural racism has a differential effect on neighborhoods based on the prevalence of White residents.^48,49,50,51,52^ We used 2017 to 2018 one-year ACS estimates of the prevalence of White residents in each residential neighborhood.^11,12,20,51,53,54,55^ We reverse coded estimates in our models so that higher-coded estimates (reflecting fewer White residents) reflected greater structural racism.

#### Economic Spatial Polarization

We assessed racialized economic spatial polarization using the index of concentrations at the extremes (race-income) (ICE-RI),^41,42,44,56,57^ calculated using 2016 to 2017 ACS data (eMethods in Supplement 1) extrapolated from the tract to the block-group level. ICE-RI estimates racialized economic segregation at the census tract level by comparing households with a White householder and annual income $100 000 or more with those with a Black householder and annual income less than $20 000.^44,58^ This measure ranges from −1 to 1, where 1 reflects population concentration in the most privileged group and −1 describes concentration in the most deprived group.^44,58^ We reverse coded the index for our models so that higher numbers reflected greater structural racism.

#### Area Deprivation

We assessed area deprivation using the area deprivation index (ADI),^59,60^ which describes concentrated socioeconomic and structural deprivation at the neighborhood level using 2015 US Census data. Greater ADI values reflect greater structural racism–mediated residential disadvantage.

### Discrete Structural Racism Indicator Variable Definitions

We characterized discrete indicators of structural racism (eAppendix 1 in Supplement 1) as those reflecting downstream sociopolitical manifestations of structurally racist residential and economic policies^4,12,42,61^ observable at the neighborhood level,^40,41,42,62^ such as reported violent crime, evictions, primary election participation, and police shootings, because several of these are not accounted for in the global measures of structural disadvantage. Data on discrete indicators were collected between 2012 and 2018, from various local and national data sources (eAppendix 1 in Supplement 1). Discrete indicators for which greater numbers reflected more structural racism included the childcare centers per square mile (collected in 2017),^63^ homes near bus stops (collected in 2017),^64^ reported violent crimes per square mile (collected in 2016),^25^ impervious areas (ie, the percentage of a region where groundwater is unable to drain because of artificial structures and pavement; collected in 2016),^65,66^ eviction rate (collected in 2017),^37^ tree cover (collected in 2016),^67^ poverty (collected in 2016),^37^ percentage unemployed (collected in 2017),^30^ percentage uninsured (collected in 2017),^30^ and police shootings (collected in 2013-2018).^68,69,70^ Discrete indicators for which greater numbers reflected less structural racism include primary election participation (collected in 2012),^71^ median household income (collected in 2017),^30^and percentage with bachelor’s degree education.^30^ We characterized race and ethnicity of neighborhoods using Census categorizations from 2010.^72^ Race and ethnicity data were reported discretely.

### Statistical Analysis

#### Descriptive Analyses

We described residential neighborhood racial distribution, age, and burden of global and discrete structural racism indicators. Due to geographic clustering, we could not assess the correlation between neighborhood characteristics and structural racism indicators through traditional methods, such as Pearson. We identified correlation through visual inspections of scatterplots (with locally estimated scatterplot smoothing), exploratory analyses, and collinearity in multivariable models. To validate these correlations, we stratified neighborhood characteristics, including discrete measures of structural racism, according to tertiles of composite structural racism indicators. For instance, we described various sociostructural features of each neighborhood as categorized by the percentage of White residents in each neighborhood.^51,53^ We estimated residential neighborhood–level disease prevalence as the number of Durham County, North Carolina, adults seen at Duke or Lincoln Community Health Center with the condition and living in a neighborhood divided by the total number of Durham County adults seen at Duke or Lincoln Community Health Center living in that residential neighborhood. We then displayed the prevalence of these estimates and global and composite measures of structural racism indicators in choropleth maps.

#### Multivariable Models

A fundamental tenet our analyses held that race is the primary sociopolitical, not biological, mediating factor through which structure influences health.^32,73,74,75,76,77,78,79,80,81^ A priori, we carefully considered that race is part of the causal pathway in the association between each structural racism indicator and outcome (ie, structural racism acts through race to exert its effects). Therefore, we did not adjust for residential neighborhood–level race in our analyses, nor did we consider race as an effect modifier in our analyses. Furthermore, we did not hypothesize an association between gender and structural racism. We attempted to address bias in this ecologic study through our careful consideration of the role of race in the causal pathway, our accounting for spatial clustering, and our separate analyses of potentially related indicators of structural racism through separate modeling strategies (eMethods in Supplement 1).

We used a Poisson distribution to model counts of each health outcome and included an offset term to account for the total population in each block group. All variables reflecting global and discrete indicators of structural racism were standardized to have mean zero and a variance of 1 to facilitate comparisons of estimates. In general, we transformed indicators as needed so that a higher value represented a greater burden of structural racism. We used separate multivariable models to quantify the association of each global and discrete structural racism indicator with the residential neighborhood prevalence of each condition (CKD, diabetes, and hypertension), while adjusting for the age of neighborhood residents (which could significantly confound the association between neighborhood residence and chronic disease prevalence) and spatial clustering of neighborhoods.

We compared 3 bayesian models with conditional autoregressive priors that account for spatial correlation and found that the model with the Leroux prior^82^ was a statistically better fit than the Besag-York-Mollie prior,^83^ while not statistically worse than the localized prior.^84^ Adjacent census block groups were identified from geographic information system shapefiles and a binary adjacency matrix was constructed that indicated which block groups were considered adjacent for all pairwise comparisons. We estimated the prevalence ratio (PR) of disease and 95% highest density interval (HDI) per 1-SD change in structural racism construct. We performed model diagnostics and then verified convergence.^85,86,87^ We also conducted sensitivity analyses to determine whether our results were affected by spatial confounding in the risk variables, which was the primary potential contributor to bias.^88^

There was no loss to follow up, and no data were missing. All structural racism indicators and outcomes constituted 48 separate models in total. A priori, we posited that global and discrete measures each reflected different manifestations of a singular structural racism construct for which we had a singular hypothesis regarding its association with neighborhood chronic condition prevalence. Accordingly, tests in 48 unique models were not independent, as we viewed all the variables as proxies for the same phenomenon of structural racism. We therefore did not adjust for multiple hypothesis testing. We performed all analyses using R software version 4.2.2 (R Project for Statistical Computing).^85,89^ Hypothesis tests were 2-sided and significance was set at α = .05. Data were analyzed between January 2021 and May 2023.

## Results

### Neighborhood Characteristics

**Figure 1.**
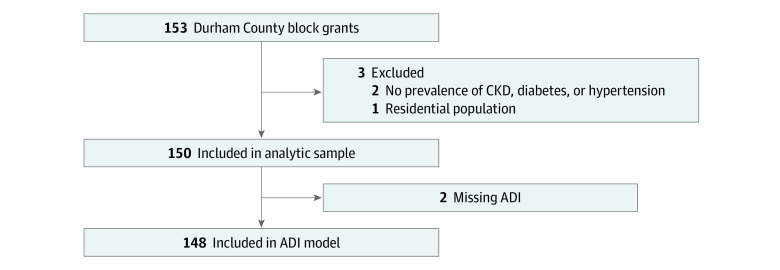
Durham County, North Carolina, Census Block Selection Flowchart ADI indicates area deprivation index; CKD, chronic kidney disease.

Among 153 total Durham County block groups, 3 were excluded for having no residential population or no observed health system encounters or outcomes. The final analytic data set included 150 residential neighborhoods (median [IQR] population, 1708 [1109-2489] residents; median [IQR] residential income, $54 531 [$37 729-$78 895]). Two block groups with missing ADI were excluded from the ADI models but were included in all other models (Figure 1). There were no other missing data in the analytic cohort. Included residential neighborhoods had median (IQR) prevalence of 2% (0%-6%) Asian residents, 30% (16%-56%) Black residents, 10% (4%-20%) Hispanic or Latino residents, 0% (0%-1%) Indigenous residents, and 44% (18%-70%) White residents (Table 1). Median (IQR) age was 35.6 (31.2-42.9) years.

**Table 1.** Residential Census Block Group Characteristics Stratified by Tertile of Percentage of White Residents in 2017

Characteristic	Median (IQR)			
	Overall	White residents tertile		
		1 (0%-26.4%)	2 (26.4%-61.3%)	3 (61.3%-97.0%)
Block groups, absolute No.	150	50	50	50
Residents per block group, No.	1708 (1109-2489)	1667 (1091-2510)	2235 (1368-3004)	1454 (973-2112)
Race and ethnicity, %				
Asian	2 (0-6)	0 (0-2)	4 (1-7)	3 (1-6)
Black	30 (16-56)	64 (51-72)	33 (25-40)	9 (5-16)
Hispanic or Latino	10 (4-20)	20 (12-28)	10 (6-16)	3 (1-9)
Indigenous	0 (0-1)	1 (0-2)	0 (0-1)	0 (0-1)
White	44 (18-70)	11 (4-18)	44 (36-53)	75 (70-83)
Racially and ethnically minoritized people^a^	56 (32-79)	87 (79-94)	56 (45-65)	23 (17-31)
Resident age, y	35.55 (31.22-42.9)	31.5 (28.5-34.8)	36.1 (31.53-42.18)	42.5 (36.52-50.38)
Discrete measures of structural racism^a^				
Childcare centers, No. per mi^2^	1.35 (0-5.04)	4.92 (0.37-7.52)	1.65 (0.41-4.55)	0 (0-1.66)
Homes near bus stops, %	69 (19-96)	98 (80-100)	64 (19-85)	26 (1-69)
Tree cover, %	50 (39-60)	42 (30-50)	48 (39-60)	58 (50-67)
Reported violent crimes, No. per mi^2^	24.84 (7.04-74.31)	91.43 (49.57-183.01)	14.57 (9.32-42.44)	4.63 (1.11-19.32)
Impervious area, %	17 (10-25)	23 (17-32)	17 (10-24)	9 (5-19)
Eviction rate, %	4 (2-7)	7 (5-9)	4 (2-6)	2 (1-4)
Primary election participation, %	40 (31-48)	26 (21-33)	41 (36-46)	50 (46-56)
Median income, $	54 531 (37 729-78 895)	34 270 (28 627-40 687)	62 310 (45 943-81 045)	76 695 (63 504-92 332)
Poverty rate, %	9 (3-24)	28 (15-41)	7 (3-16)	4 (0-7)
Bachelor’s degree, %	25 (16-32)	14 (9-22)	26 (19-34)	30 (26-37)
Unemployed, %	4 (2-6)	5 (4-8)	4 (2-6)	2 (1-4)
Uninsured, %	13 (7-28)	29 (18-41)	13 (9-20)	5 (3-8)
Police shootings, No. (%)	7 (4.67)	5 (10)	2 (4)	0

^a^ Racial and ethnically minoritized people refers to the percentage of the total population reporting their race on the census as any option besides White and/or their ethnicity to be Hispanic or Latino. Indigenous in this study reflects American Community Survey and Census determinations of individuals who reported American Indian or Alaska Native alone or in combination with 1 or more races. The top 4 tribal affiliations reported by Durham County residents in 2019 were Cherokee, Chippewa, Navajo, and Sioux.

### Neighborhood Burden of Global and Discrete Measures of Structural Racism

The neighborhood-level burden of discrete structural racism indicators corresponded with the burden of global indicators of structural racism. For instance, neighborhoods classified into the lowest tertile of percentage of White residents had the highest median number of reported violent crimes. There was a similar pattern for several other discrete structural racism indicators (Table 1). We observed similar correspondences between ICE-RI and the ADI and discrete indicators (eTable in Supplement 1).

### Structural Racism and Neighborhood Health

**Figure 2.**
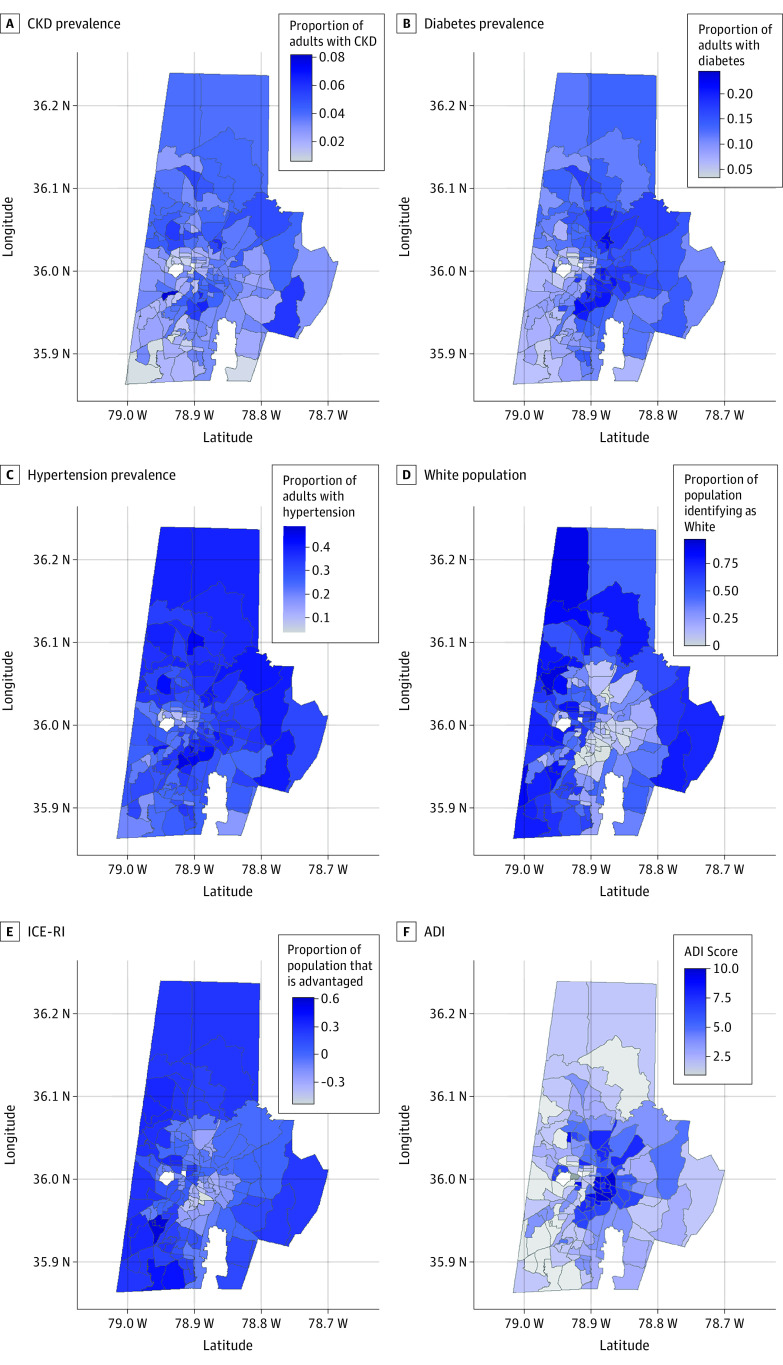
Geospatial Maps Depicting Prevalences in Durham County, North Carolina, Neighborhoods ADI indicates area deprivation index; CKD, chronic kidney disease; and ICE-RI, index of concentrations at the extremes (race-income).

Neighborhood prevalence of chronic conditions varied for CKD (0.6% to 8.2%), diabetes (3.3% to 24.4%), and hypertension (3.6% to 48.9%). There were distinct clusters of neighborhoods with high condition prevalence (Figure 2). Residential neighborhoods with the lowest prevalence of CKD, diabetes, and hypertension had higher median proportions of White residents, higher concentrations of White households with income $100 000 or greater, and lower area deprivation. Similarly, neighborhoods with the lowest prevalence of CKD, diabetes, and hypertension had the lowest median rates of poverty and the highest median rates of college education (Table 2).

**Table 2.** Composite and Discrete Measures of Neighborhood Structural Racism Stratified by Tertiles of Neighborhood Prevalence Chronic Kidney Disease, Diabetes, and Hypertension

Characteristic	Tertile prevalence, median (IQR)								
	Chronic kidney disease			Diabetes			Hypertension		
	1 (0.6%-2.8%)	2 (2.8%-3.9%)	3 (3.9%-8.2%)	1 (3.3%-10.0%)	2 (10.0%-15.3%)	3 (15.3%-24.4%)	1 (3.6%-29.0%)	2 (29.0%-35.6%)	3 (35.6%-48.9%)
Block groups, No.	50	50	50	50	50	50	50	50	50
White population, %^a^	54 (34 to 68)	34 (15 to 57)	43 (9 to 73)	67 (53 to 79)	51 (35 to 70)	12 (4 to 25)	56 (34 to 67)	40 (21 to 67)	34 (10 to 72)
ICE-RI^a^	0.17 (−0.01 to 0.29)	0.02 (−0.14 to 0.23)	0.03 (−0.21 to 0.29)	0.25 (0.10 to 0.33)	0.15 (0.03 to 0.28)	−0.16 (−0.27 to 0.00)	0.17 (0.01 to 0.28)	0.06 (−0.04 to 0.28)	0.04 (−0.21 to 0.29)
ADI	2 (2 to 4)	4 (2 to 7)	4 (2 to 7)	2 (1 to 3)	3 (2 to 4)	7 (5 to 9)	2 (2 to 4)	4 (2 to 7)	4 (2 to 8)
Homes near bus stops, %	84 (36 to 96)	63 (24 to 95)	43 (1 to 97)	69 (33 to 91)	26 (0 to 83)	89 (59 to 100)	88 (67 to 97)	54 (17 to 89)	43 (1 to 95)
Tree cover, %	44 (35 to 56)	48 (40 to 57)	54 (37 to 63)	51 (38 to 60)	52 (42 to 63)	43 (35 to 54)	44 (33 to 55)	50 (40 to 59)	52 (40 to 63)
Reported violent crimes, No. per mi^2^	28.44 (11.20 to 62.95)	18.67 (8.65 to 80.09)	21.56 (1.95 to 89.89)	18.54 (3.48 to 50.12)	10.5 (2.09 to 46.48)	68.32 (25.74 to 183.01)	35.92 (13.18 to 64.95)	14.99 (7.34 to 80.17)	16.52 (2.88 to 75.15)
Impervious area, %	20 (13 to 28)	17 (10 to 23)	13 (6 to 25)	18 (10 to 26)	15 (5 to 21)	19 (12 to 29)	23 (14 to 28)	16 (10 to 24)	13 (5 to 21)
Eviction rate, %	4 (2 to 6)	4 (2 to 6)	4 (2 to 8)	3 (1 to 5)	3 (2 to 5)	7 (5 to 9)	3 (2 to 5)	5 (3 to 9)	5 (2 to 7)
Primary election participation, %	40 (33 to 48)	37 (30 to 46)	42 (28 to 50)	48 (36 to 56)	44 (36 to 49)	31 (23 to 37)	38 (31 to 48)	40 (32 to 48)	43 (28 to 49)
Median income, $	62 891 (40 037 to 80 486)	51 738 (39 347 to 78 895)	54 583 (34 270 to 77 031)	65 504 (50 320 to 85 226)	70 646 (43 386 to 82 485)	37 213 (28 998 to 51 149)	56 793 (42 831 to 83 750)	60 328 (37 228 to 83 263)	55 729 (37 120 to 77 917)
Poverty rate, %	8 (2 to 22)	9 (3 to 25)	4 (0 to 12)	7 (3 to 14)	22 (9 to 38)	9 (3 to 22)	7 (2 to 22)	12 (4 to 25)	9 (3 to 25)
Bachelor’s degree, %	28 (23 to 35)	22 (13 to 32)	19 (1 to 29)	3 (27 to 37)	25 (21 to 34)	12 (8 to 21)	28 (23 to 34)	24 (15 to 32)	18 (12 to 27)
Unemployed, %	4 (2 to 5)	4 (2 to 6)	3 (1 to 4)	4 (2 to 6)	5 (3 to 8)	3 (2 to 5)	3 (2 to 4)	4 (2 to 5)	4 (2 to 6)
Uninsured, %	11 (5 to 25)	14 (6 to 23)	8 (3 to 13)	10 (7 to 19)	28 (16 to 38)	10 (5 to 21)	12 (7 to 28)	12 (5 to 23)	14 (6 to 23)
Police shootings, No. (%)	1 (2)	4 (8)	2 (4)	1 (2)	1 (2)	5 (10)	1 (2)	3 (6)	3 (6)

Abbreviations: ADI, area deprivation index; ICE-RI, index of concentrations at the extremes (race-income).

^a^ Percentage of White population increases and increased values of ICE-RI reflect greater structural advantage.

In models, greater burdens of global structural racism indicators were associated with higher prevalences of CKD, diabetes, and hypertension. For instance, each 1-SD decrease in neighborhood percentage White population, a marker of increasing social disadvantage, was associated with higher prevalences of CKD (PR, 1.27 [95% CI, 1.18-1.35]), diabetes (PR, 1.43 [95% HDI, 1.37-1.52]), and hypertension (PR, 1.19 [95% HDI, 1.14-1.25]). There were similar associations of ICE-RI and ADI with neighborhood prevalence (Table 3). Greater burdens of most discrete indicators of structural racism were also associated with greater neighborhood prevalences of CKD, diabetes, and hypertension. For instance, each 1-SD increase in neighborhood violent crime reporting was associated with increased prevalences of CKD (PR, 1.15 [95% HDI, 1.07-1.23]), diabetes (PR, 1.20 [95% HDI, 1.13-1.28]), and hypertension (PR, 1.08 [95% HDI, 1.02-1.14]). Similarly, each 1-SD decrease in neighborhood income was associated with higher prevalences of CKD (PR, 1.19 [95% HDI, 1.12 to 1.28]), diabetes (PR, 1.25 [95% HDI, 1.18 to 1.33]), and hypertension (PR, 1.08 [95% HDI, 1.03 to 1.14]) (Table 3). There was no evidence that these results were sensitive to spatial confounding.

**Table 3.** Association of Composite and Discrete Structural Racism Constructs With Neighborhood Chronic Kidney Disease, Diabetes, and Hypertension Prevalence, Adjusted for Median Age of Residential Neighborhood Population and Spatial Correlation

Measure	Estimated adjusted prevalence ratio (95% highest density interval)^a^		
	Chronic kidney disease	Diabetes	Hypertension
**Composite measures of structural racism**			
Percentage of White population, per 1-SD decrease	1.27 (1.18-1.35)	1.43 (1.37-1.52)	1.19 (1.14-1.25)
White ≥$100 000 ICE-RI, per 1-SD decrease	1.27 (1.20-1.35)	1.35 (1.28-1.43)	1.14 (1.09-1.19)
ADI	1.25 (1.18-1.32)	1.35 (1.30-1.43)	1.15 (1.10-1.19)
**Discrete measures of structural racism**			
Child care centers	1.10 (1.03-1.17)	1.14 (1.07-1.22)	1.08 (1.03-1.13)
Homes near bus stops	1.05 (0.97-1.14)	1.08 (0.99-1.17)	0.97 (0.92-1.03)
Tree cover, per 1-SD decrease	1.04 (0.96-1.12)	1.04 (0.96-1.12)	0.96 (0.92-1.01)
Violent crimes	1.15 (1.07-1.23)	1.20 (1.13-1.28)	1.08 (1.02-1.14)
Impervious area	1.01 (0.94-1.09)	0.99 (0.92-1.07)	0.93 (0.88-0.98)
Eviction rate	1.09 (1.02-1.17)	1.14 (1.07-1.22)	1.07 (1.02-1.12)
Primary election participation, per 1-SD decrease	1.15 (1.06-1.23)	1.32 (1.23-1.41)	1.06 (1.01-1.14)
Median household income, per 1-SD decrease	1.19 (1.12-1.28)	1.25 (1.18-1.33)	1.08 (1.03-1.14)
Poverty rate	1.14 (1.06-1.22)	1.23 (1.15-1.31)	1.07 (1.02-1.13)
Percentage with Bachelor’s degree, per 1-SD decrease	1.22 (1.15-1.3)	1.3 (1.23-1.37)	1.16 (1.12-1.22)
Percentage unemployed	1.09 (1.02-1.16)	1.15 (1.08-1.22)	1.06 (1.01-1.11)
Percentage uninsured	1.13 (1.05-1.21)	1.24 (1.17-1.32)	1.10 (1.05-1.16)
Police shootings	1.01 (0.95-1.08)	1.06 (0.99-1.13)	1.02 (0.98-1.07)

Abbreviations: ADI, area deprivation index; ICE-RI, index of concentrations at the extremes (race-income).

^a^ Relative difference in the prevalence of each chronic disease per each 1-SD increase in structural racism construct (except where designated as relative difference per 1-SD decrease). Relative differences greater than 1.0 reflect greater prevalence for each standard deviation increase in structural racism construct. Estimates in each cell derived from a different model (48 unique models depicted).

## Discussion

In this cross-sectional ecologic study of residential neighborhoods in Durham County, North Carolina, we found that an increased burden of structural racism, measured through multiple global and discrete indicators, was consistently associated with increased residential neighborhood prevalences of multiple chronic conditions for which racial disparities are highly prevalent. Global and discrete indicators of structural racism were correlated, and the directions of their associations with neighborhood prevalence of health conditions were highly consistent. Discrete indicators of violent crime reporting, eviction rates, primary election voting, income, education, unemployment, and uninsurance were all statistically significantly associated with neighborhood prevalence of CKD, diabetes, and hypertension, suggesting these indicators may reflect important factors to consider in efforts to develop equity-promoting community health interventions.^12,41^

To our knowledge, prior studies have not investigated the associations of both global and discrete indicators of structural racism with residential neighborhood prevalence of chronic illnesses for which racially and ethnically minoritized individuals experience significant health inequities. Our findings corroborate and extend insights from previous work demonstrating associations of structural disadvantage with health, and they uniquely illustrate strong, concurrent associations of neighborhood health inequities with social conditions mediated through racialized policies and practices. These new insights help to underscore the critical importance of work, including research, to identify and intervene on social and environmental determinants of health as efforts to improve health equity continually evolve.

By using local data to characterize some discrete structural racism constructs (eg, evictions and voter participation), this study expands insight regarding the potential influence of local social and political contexts on residential neighborhood health. Efforts to further understand the mechanisms through which these local factors (which have not previously been widely studied with regard to their associations with health) might influence neighborhood health are warranted.

### Limitations

This study has some limitations. First, this was a cross-sectional analysis, from which causal inferences cannot be drawn. Furthermore, as this is an ecological study, we cannot draw inferences regarding individual health from these study findings, which may be subject to ecological fallacy and bias. Second, many of the structural racism indicators we used were not originally developed to describe structural racism. Therefore, our constructs may fail to capture the full multidimensional and nuanced essence and impacts of structural racism.^12,47,90^ While it is a strength that we assessed associations between global and discrete structural racism indicators and health through multiple unique data sources, the sources from which these data emerged were not harmonized for research. For instance, our varied data sources provided information over different time periods, which could affect their precision relative to our ascertainment of neighborhood health. Third, while our use of electronic health record data to characterize residential neighborhood disease prevalence likely improved the specificity of our measures, prevalence data were ascertained from approximately 85% of Durham County residents and only were available for those who used health care. This could introduce imprecision in our estimates. For instance, prevalence estimates could underestimate the magnitude of associations among neighborhoods for which health care utilization might be low. Additionally, we studied a small number of residential neighborhoods within a single county in North Carolina. Findings may differ in other areas of the US with differing populations, geographical characteristics, or sociopolitical contexts.

## Conclusions

This cross-sectional study found numerous indicators of structural racism associated with inequities in residential neighborhood health. Although caution should be used when interpreting findings from this cross-sectional ecological analysis, these structural racism constructs could be considered in future efforts to mitigate neighborhood health inequities.

## Notes

Supplement 1.
eMethods.
**eTable.** Stratified Census Block Group Characteristics **eAppendix 1.** Indicators of Structural Racism **eAppendix 2.** Structural Racism Variables, Direction Associated With More Structural Racism, and Association With Structural Racism
eReferences.

Supplement 2.
Data Sharing Statement
