# Supplementary material for: Residential Structural Racism and Prevalence of Chronic Health Conditions
Source: JAMA Netw Open. 2023 Dec 21;6(12):e2348914. doi: 10.1001/jamanetworkopen.2023.48914 (PMC10739116; doi:10.1001/jamanetworkopen.2023.48914)
Supplement: Supplement 1. — eMethods. eTable. Stratified Census Block Group Characteristics eAppendix 1. Indicators of Structural Racism eAppendix 2. Structural Racism Variables, Direction Associated With More Structural Racism, and Association With Structural Racism eReferences. [file jamanetwopen-e2348914-s001.pdf]

Supplemental Online Content

Mohottige D, Davenport CA, Bhavsar N, et al. Residential structural racism and prevalence of chronic health conditions. *JAMA Netw Open*. 2023;6(12):e2348914. doi:10.1001/jamanetworkopen.2023.48914

eMethods.
eTable. Stratified Census Block Group Characteristics
eAppendix 1. Indicators of Structural Racism
eAppendix 2. Structural Racism Variables, Direction Associated With More Structural Racism, and Association With Structural Racism
eReferences.

This supplemental material has been provided by the authors to give readers additional information about their work.

© 2023 Mohottige D et al. *JAMA Network Open.*

eMethods

Exploratory analyses. We assessed for spatial correlation between the block groups by first fitting separate Poisson generalized linear models regressing CKD prevalence on each structural racism indicator and age, with an offset term to account for population size in the block group. We identified adjacent census block groups using GIS shapefiles and constructed a binary matrix indicating pairwise adjacent block groups. We measured spatial correlation using Moran's I based on this adjacency matrix and the residuals from the Poisson models and found statistically significant evidence of underlying spatial correlation in all models. We also identified significant collinearity when attempting to mutually adjust for structural racism indicators. The results of these exploratory analyses led us to our final, minimally adjusted modeling approach described below. Our primary effort to address bias was to explore the presence of spatial correlation between block groups, perform statistical tests to identify significant correlation, and adjust our approach accordingly.

Due to geographic clustering, we could not assess the correlation between neighborhood characteristics and structural racism indicators through traditional methods such as Pearson's. We identified correlation through visual inspections of scatterplots (with LOESS), exploratory analyses, and collinearity in multivariable models. To validate these correlations, we stratified neighborhood characteristics, including discrete measures of structural racism, according to tertiles of composite structural racism indicators.

Multivariable models. In our primary analyses, we quantified associations of both global and discrete structural racism indicators with residential neighborhood health defined as prevalence of CKD, DM, and HTN, respectively, while adjusting for the median age of neighborhood residents (which could significantly confound the relation between neighborhood residence and chronic disease prevalence) and spatial clustering of neighborhoods. We did not adjust for neighborhood residential racial composition in our analyses, as we carefully considered that race would be the primary mediator of any relationship observed between structural racism indicators (e.g., income, eviction rates) and neighborhood health.

We compared three different Bayesian hierarchical models with conditional autoregressive (CAR) priors for the spatial random effects to account for spatial correlation/clustering; the priors considered were Besag-York-Mollie (BYM), Leroux, and localized. We used a Poisson distribution to model the counts of each health outcome and included an offset term to account for the total population in each block group. We fit the models using Markov chain Monte Carlo algorithm to estimate the posterior distribution and compared the models using WAIC and convergence criteria. We found that the model with the Leroux prior[86] was a statistically better fit than the BYM prior, but not statistically worse than the localized prior. All variables reflecting global, and discrete indicators of structural racism were standardized to have mean zero and a variance of one to facilitate comparisons of estimates. We fit the model separately for each global and discrete structural racism construct and estimated the prevalence ratio of disease (per 1 standard deviation increase in structural racism construct) and 95% highest density interval (HDI). We performed model diagnostics and then verified convergence. [89-91] We also conducted sensitivity analyses to determine if our findings were influenced by spatial confounding in the variables.[92]

Assessment of Chronic Disease Prevalence

We characterized census block groups according to the population prevalence of three common chronic diseases (chronic kidney disease, diabetes, and hypertension) of adults defined as aged 18 and older who were residents within each census block group. Ensuring privacy was a

© 2023 Mohottige D et al. *JAMA Network Open.*

paramount issue so all metrics in the community compass were aggregated at the level of block groups, and these data were masked if there were less than 3 individuals in a block group in our effort to protect patient privacy. Each chronic condition noted below was determined based on lab values/health encounters for patients in the calendar year specified[23], who also had a recorded Durham County physical home address or post office box. If patients were hospitalized across multiple days across several calendar years, the discharge date was used to determine whether they would be included within that end-date calendar year. Using the EHR data and input from subspecialty experts, we defined the following conditions:

Chronic kidney disease Counts of adults with Kidney Disease Improving Global Outcomes (KDIGO)-defined CKD III-V were ascertained based on lab values and health encounters in 2017[23]. We considered individuals as having CKD III-V if they had at least 2 eGFR measurements less than 30 ml/min/1.73m^2 (by CKD-EPI)[96,97] at least 90 days apart, or one GFR less than 30 and one albumin to creatinine ratio greater than or equal to 30 mg/g. We did not exclude individuals receiving any form of kidney replacement therapy (i.e., hemodialysis or peritoneal dialysis) or kidney transplant as these data were not available. Notably, our analyses included eGFR as calculated with the Black race modifier because of the timing of the National Kidney Foundation-American Society of Nephrology [98]joint taskforce recommendation for removal of the race modifier, which did not go into effect at Duke until June 2021[98-100]. We categorized neighborhood prevalence of each chronic disease into tertiles.

Diabetes The number of adult residents with diabetes (DM) was assessed in 2017. DM was defined as any individual who had type II diabetes according to either ICD codes 249, 250, Eo8, E09, E11, or E13 or based on a hemoglobin A1C (HbA1C) measurement of 6.4% or above.

Hypertension The total number of individuals with hypertension (HTN) in each block group was ascertained in 2018. HTN was defined as any individual who had a systolic blood pressure >140 mmHg or diastolic blood pressure > 90 mmHg within a 12-month period excluding inpatient readings and/or an ICD 9 code of 401/405, and ICD 10 codes of I10, or I15. This categorization excluded HTN in pregnancy. We did not have data collected and reported on the Compass regarding the HTN data in 2017, thus the first time point of availability of this data was in 2017.

© 2023 Mohottige D et al. *JAMA Network Open.*

eTable. Stratified Census Block Group Characteristics

Table 1a
Census block group characteristics stratified by **tertiles of ICE race-income** in 2017 (not age adjusted). Values given are median [IQR] except police shootings which is n (%). * ICE-RI is not reverse coded in this table

**Block groups (Absolute number)**	Overall<br>150	ICE-RI Tertile 1 [-0.533,-0.0484)<br>50	ICE-RI Tertile 2 [-0.0484,0.234)<br>48	ICE-RI Tertile 3 [0.234,0.612]<br>52
**Residents per block group (median number [IQR**])**	1708 [1108.75-2488.5]	1561.5 [1036.5-2427]	2077.5 [1346.5-2787]	1694.5 [1162.75-2394.75]
**Race and Ethnicity (median %)**				
Black and African American	0.3 [0.16-0.56]	0.61 [0.38-0.71]	0.35 [0.25-0.46]	0.12 [0.07-0.21]
White	0.44 [0.18-0.7]	0.11 [0.04-0.19]	0.41 [0.32-0.55]	0.73 [0.62-0.82]
Asian	0.02 [0-0.06]	0 [0-0.06]	0.02 [0-0.06]	0.02 [0.01-0.06]
Hispanic and Latino	0.1 [0.04-0.2]	0.19 [0.1-0.28]	0.11 [0.04-0.22]	0.05 [0.02-0.09]
Indigenous	0 [0-0.01]	0.01 [0-0.01]	0 [0-0.01]	0 [0-0.01]
Racially and ethnically minoritized people*	0.56 [0.32-0.79]	0.87 [0.77-0.94]	0.58 [0.41-0.71]	0.25 [0.17-0.4]
**Median Age (years)**	35.55 [31.22-42.9]	31.3 [27.98-35.22]	34.8 [31.6-38.4]	42.6 [37.2-50]
**Discrete Measures of Structural Racism**				
Childcare centers (N per square mile)	1.35 [0-5.04]	4.36 [0.2-7.45]	2.44 [0.41-5.02]	0.1 [0-1.34]
Homes near bus stops (%)	0.69 [0.19-0.96]	0.99 [0.8-1]	0.69 [0.36-0.91]	0.1 [0-0.44]
Tree Cover (%)	0.5 [0.39-0.6]	0.41 [0.29-0.49]	0.45 [0.38-0.55]	0.61 [0.54-0.69]
Reported Violent crimes (N per square mile)	24.84 [7.04-74.31]	91.43 [46.83-183.01]	20.24 [10.49-54.33]	4.31 [0.99-11.8]
Impervious area (%)	0.17 [0.1-0.25]	0.25 [0.19-0.33]	0.18 [0.12-0.24]	0.08 [0.05-0.14]
Eviction rate (%)	0.04 [0.02-0.07]	0.07 [0.05-0.09]	0.04 [0.03-0.06]	0.02 [0.01-0.04]
Primary election participation (%)	0.4 [0.31-0.48]	0.26 [0.21-0.33]	0.41 [0.35-0.46]	0.5 [0.46-0.54]
Median Income ($)	54531 [37729.25-78895.25]	33455.5 [28627.25-39850.25]	52091 [43879.75-73473.5]	80429.5 [68354-94670.5]
Poverty rate (%)	0.09 [0.03-0.24]	0.28 [0.15-0.42]	0.09 [0.04-0.18]	0.03 [0-0.06]
Bachelor’s degree (%)	0.25 [0.16-0.32]	0.16 [0.09-0.22]	0.23 [0.16-0.31]	0.3 [0.26-0.36]
Unemployed (%)	0.04 [0.02-0.06]	0.05 [0.03-0.08]	0.04 [0.02-0.07]	0.03 [0.02-0.04]
Uninsured (%)	0.13 [0.07-0.28]	0.27 [0.18-0.41]	0.15 [0.09-0.29]	0.06 [0.02-0.09]
Police shootings	7 (4.67)	5 (10)	2 (4.17)	0 (0)

© 2023 Mohottige D et al. *JAMA Network Open.*

	Overall	ICE-RI Tertile 1 [-0.533,-0.0484)	ICE-RI Tertile 2 [-0.0484,0.234)	ICE-RI Tertile 3 [0.234,0.612]
**Census tracts (absolute number)**	57	20	19	18
**Residents per block census tract (median number [IQR**])**	1877.85 [1261.45-2771.2]	1696.13 [1092.51-2525.08]	2291.17 [1365.98-2973.5]	1823.02 [1248.57-2488.46]
**Race and Ethnicity (median %)**				
Black and African American	0.3 [0.16-0.54]	0.59 [0.32-0.68]	0.36 [0.26-0.5]	0.12 [0.07-0.22]
White	0.43 [0.2-0.7]	0.12 [0.04-0.2]	0.38 [0.31-0.53]	0.73 [0.6-0.82]
Asian	0.02 [0-0.07]	0 [0-0.07]	0.02 [0-0.06]	0.02 [0.01-0.07]
Hispanic and Latino	0.09 [0.03-0.2]	0.18 [0.1-0.29]	0.1 [0.04-0.23]	0.03 [0.02-0.09]
Indigenous	0 [0-0.01]	0 [0-0.01]	0 [0-0.01]	0 [0-0.01]
Racially and ethnically minoritized people*	0.56 [0.3-0.79]	0.86 [0.77-0.93]	0.59 [0.44-0.72]	0.25 [0.17-0.4]
**Median Age (years)**	36.66 [31.24-44.07]	31.05 [27.62-35.1]	34.92 [32.62-40.05]	43.22 [37.53-50.52]
**Discrete Measures of Structural Racism**				
Childcare centers (N per square mile)	1.05 [0-4.57]	2.7 [0-6.37]	2.13 [0.23-4.86]	0.02 [0-1.08]
Homes near bus stops (%)	0.54 [0.1-0.89]	0.96 [0.74-1]	0.49 [0.2-0.85]	0.05 [0-0.33]
Tree Cover (%)	0.51 [0.41-0.6]	0.42 [0.31-0.5]	0.47 [0.38-0.55]	0.62 [0.56-0.7]
Violent crime reporting (N per square mile)	14.58 [4.69-54.15]	61.8 [34.98-125.08]	12.84 [8.32-45.46]	2.99 [0.67-9.94]
Impervious area (%)	0.16 [0.08-0.23]	0.22 [0.17-0.28]	0.16 [0.1-0.23]	0.08 [0.03-0.13]
Eviction rate (%)	0.04 [0.02-0.07]	0.06 [0.04-0.09]	0.04 [0.03-0.06]	0.02 [0.01-0.04]
Primary election participation (%)	0.4 [0.31-0.48]	0.27 [0.22-0.33]	0.39 [0.34-0.46]	0.5 [0.45-0.54]
Median Income ($)	61925.67 [39484.21-80580.1]	35394.45 [29271.94-40897.2]	54082.17 [45171.76-78853.11]	80457.19 [68031.16-95327.95]
Poverty rate (%)	0.08 [0.02-0.2]	0.24 [0.12-0.4]	0.08 [0.03-0.16]	0.03 [0-0.05]
Bachelor's degree (%)	0.25 [0.16-0.33]	0.16 [0.08-0.25]	0.23 [0.17-0.31]	0.3 [0.26-0.35]
Unemployed (%)	0.03 [0.02-0.06]	0.05 [0.03-0.07]	0.04 [0.01-0.07]	0.03 [0.02-0.04]
Uninsured (%)	0.12 [0.06-0.25]	0.24 [0.17-0.4]	0.13 [0.09-0.29]	0.06 [0.01-0.08]
Police shootings	7 (12.28)	5 (25.00)	2 (10.53)	0 (0)

© 2023 Mohottige D et al. *JAMA Network Open.*

Supplemental Table 1b
Census block group characteristics stratified by **tertiles of Area Deprivation Index** in 2017 (not age adjusted). Values given are median [IQR] except police shootings which is n (%).

	Overall	ADI Tertile 1 [1,6)	ADI Tertile 2 [6,9)	ADI Tertile 3 [9,10]
**Block groups (Number of block groups)**	148	42	51	55
**Residents (N)**	1728 [1162.75-2498]	1394 [1073.25-1932.5]	2118 [1225.5-3043]	1864 [1260-2409.5]
**Race and Ethnicity (%)**				
Black and African American	0.31 [0.16-0.56]	0.61 [0.45-0.75]	0.37 [0.24-0.54]	0.12 [0.07-0.23]
White	0.43 [0.18-0.7]	0.1 [0.03-0.19]	0.4 [0.3-0.57]	0.71 [0.59-0.82]
Asian	0.02 [0-0.06]	0 [0-0.01]	0.01 [0-0.07]	0.04 [0.01-0.08]
Hispanic and Latino	0.1 [0.03-0.2]	0.2 [0.12-0.3]	0.1 [0.06-0.19]	0.04 [0.02-0.09]
Indigenous	0 [0-0.01]	0.01 [0-0.02]	0 [0-0.01]	0 [0-0.01]
Racially and ethnically minoritized people*	0.56 [0.31-0.79]	0.87 [0.79-0.95]	0.58 [0.41-0.71]	0.27 [0.19-0.42]
**Median Age (years)**	35.85 [31.3-42.95]	32.75 [28.98-38.62]	35.3 [31.3-42.4]	39.9 [34.5-47.55]
**Discrete Measures of Structural Racism**				
Childcare centers (N per square mile)	1.35 [0-5.02]	5.24 [1.46-8.29]	0.8 [0-3.45]	0.51 [0-3.89]
Homes near bus stops (%)	0.69 [0.18-0.96]	0.99 [0.84-1]	0.56 [0.22-0.87]	0.32 [0.01-0.71]
Tree Cover (%)	0.5 [0.39-0.6]	0.4 [0.3-0.5]	0.49 [0.42-0.59]	0.57 [0.44-0.66]
Violent crimes (N per square mile)	24.84 [6.91-75.63]	91.43 [58.05-194.06]	17.13 [8.63-47.79]	5.1 [1.24-25.93]
Impervious area (%)	0.17 [0.1-0.25]	0.24 [0.18-0.32]	0.17 [0.1-0.24]	0.1 [0.05-0.19]
Eviction rate (%)	0.04 [0.02-0.07]	0.07 [0.05-0.1]	0.03 [0.02-0.07]	0.03 [0.02-0.04]
Primary election participation (%)	0.4 [0.31-0.49]	0.26 [0.22-0.32]	0.4 [0.35-0.46]	0.5 [0.44-0.56]
Median Income ($)	54600.5 [38345-78980.5]	31398.5 [27975.25-38143]	54583 [47759-72452]	80179 [65503.5-96148.5]
Poverty rate (%)	0.09 [0.03-0.24]	0.31 [0.2-0.41]	0.07 [0.04-0.15]	0.03 [0-0.08]
Bachelor’s degree (%)	0.25 [0.16-0.32]	0.12 [0.08-0.2]	0.25 [0.19-0.33]	0.3 [0.27-0.37]
Unemployed (%)	0.04 [0.02-0.06]	0.05 [0.04-0.08]	0.04 [0.02-0.07]	0.03 [0.02-0.04]
Uninsured (%)	0.13 [0.07-0.28]	0.3 [0.22-0.39]	0.14 [0.09-0.2]	0.06 [0.03-0.1]
Police shootings	7 (4.73)	5 (11.9)	1 (1.96)	1 (1.82)

© 2023 Mohottige D et al. *JAMA Network Open.*

eAppendix 1: Indicators of structural racism
We carefully considered the many domains through which structural racism operates, which are described below, along with our considerations for each variable used.

BUILT ENVIRONMENT
Impervious areas (Durham Compass)
The built environment was characterized using a 2016 measure of impervious areas, an Environmental Protection Agency variable intended to capture the percent of a region where groundwater is unable to drain because of artificial structures and pavement. Greater percentages of impervious land area may occur in areas manifesting greater structural racism. Furthermore, more impervious areas may cause environmental harm and poor health outcomes via impaired water quality (pollutant accumulation may be then washed into streams), dis-regulated water quantity (impervious areas prevent natural groundwater accumulation and favors storm drainage accumulation, which may cause flooding etc.), and via temperature (rainwater in impervious areas may become extremely heated, thus disrupting the natural ecosystems of the streams into which they drain).[68,101]
Household proximity to bus stops (Durham Compass)
We assessed access to public transport options through a measure of proximity to bus stops determined by Go Triangle transit and city-council GIS in 2017. Access was defined as residential units within ¼ mile Euclidean distance to a bus stop. Lower transportation access has been associated with increased barriers to health care access.[102] However, greater bus and other public transit access may be located in more minoritized/marginalized communities in certain contexts, including in Durham County wherein a greater proportion of White individuals are car owners.
Tree coverage (Durham Compass)
This measure of tree coverage was ascertained from the National Agricultural Imagery Program (NAIP) data sourced in 2016 from the EPA EnviroAtlas for urbanized parts of Durham County. Greater tree canopy has been associated with communities characterized by greater sociopolitical advantage. Greater tree coverage has also been associated with multiple improved health outcomes, mediated by greater physical activity and social cohesion.[103]

ECONOMICS
Median household income (Durham Compass)
The median household income of the neighborhood in dollars was assessed in 2017-2018, and represent a marker of economic stability, which are impacted by structural racism and associated policies. Lower median household income has been associated with greater prevalence of CKD, DM, and HTN.[104,105]
Poverty rates (Eviction Lab)
Poverty rates were determined from the Eviction Lab in 2016 using 2014-2018 5-Year American Community Survey estimates. Communities with a greater proportion of minoritized individuals are characterized by greater poverty due to structural disadvantage and structural racism. Poverty has been associated with greater prevalence of CKD, DM, and HTN.[3,104,106,107]

HOUSING
Eviction rates (Eviction Lab)
Evictions were measured in 2017-2018 by determining the number of homes that received an eviction judgment in which renters were ordered to leave. This value counts only single addresses (and nor all persons) that received this judgment over a denominator of the number of occupied renting households in each area. Information on the number of renter homes in an area comes from the U.S. Census and ESRI Business analyst demographic estimates.

© 2023 Mohottige D et al. *JAMA Network Open.*

Eviction rates have been described as a key driver of health outcomes because of the legal consequences of having had an eviction including subsequent exposure to a higher risk rental market, increased risks of homelessness/housing insecurity, and interrupted health care engagement if physical distance and transportation is a barrier.[108,109] Several prior studies have described the disproportionate impact of evictions on racially and ethnically minoritized groups across multiple cities, a disadvantage that may be influenced by individual level and neighborhood level and social network level features.[108]When numerous evictions are concentrated in a specific neighborhood, individuals living within that area may be adversely impacted because of this state of instability, which does not guarantee afforded privileges of wealthy individuals. This may also diminish civic empowerment and policy changes that are health promoting for those who are displaced. Evictions have also been described as a proxy for gentrification-led displacement of individuals.[110]

POLICING CRIMINALIZATION AND SAFETY

Violent crime reporting (Durham Compass)

Violent crime reporting was assessed in 2016 and described as the number of incidents per square mile, as reported to the Durham Police department by callers.
The excess criminalization of Black and other minoritized people has been described over decades of research and results from a complex amalgam of structural and interpersonal racism, which impact the adjudication of incidents. The downstream impact of this disproportionate penalization has had numerous impacts including by restricting job access and associated health benefits, access to public housing in some cases, access to foods stamps and other benefits, licenses/work permits, financial support for higher education, and voting rights in many states, even after a conviction is served.[111] Communities with greater exposure to violent crime reporting are more likely to live in structurally disadvantaged neighborhoods, with downstream impacts on a range of health outcomes. Individuals exposed to greater violent crime reporting may have increased vulnerability to accidental violence/mortality, greater perceptions of danger, less perceptions of safety necessary to engage in health-promoting behaviors like physical exercise, and less perceived social capital.[112] Increased allostatic load or increased stress, anxiety, depression associated with living in this type of environment may also be a mediating factor in this relationship between health and violent crime reporting.[113]

Police shooting (Mapping Police Violence Database)

Police shootings (including those of on duty police officers, incidents when a person is killed via use of a chokehold, baton, taser or other means, as well as killings by off-duty police) were assessed from 2013-2018 using data from mappingpoliceviolence.org and we identified neighborhoods in which a shooting occurred using block group FIPS codes mapped onto the locations identified within this data source. The disproportionate criminalization of Black and other minoritized individuals by state sanctioned policies has been described long before the murder of George Floyd.[114-118] Racially minoritized individuals in the U.S.- especially Black individuals- have long experienced disproportionate exposure to police violence and fatal police shootings, which are a public health emergency.[47,72,115,116,119] The impacts of this harm include years of life lost, morbidity in terms of anxiety and severe mental health consequences.[116]

POLITICAL ENGAGEMENT

Primary election voting among active voters (Durham Compass)

Primary election participation was assessed in 2012 as the proportion of active voters who voted in the primary election that year using data from the Durham County Board of Elections. Social capital has been defined as a critical feature of social structures "and [includes] interpersonal trust and norms of reciprocity and mutual aid, which act as resources for individuals and facilitate social action."[120] Associations between social capital and political activities like voting are well described and demonstrate how geographic variations in voter

turnout has been linked to civic trust and social capital, as defined above. Furthermore, voter participation and engagement is influenced by racism and other social forces via 1) voter disenfranchisement of disproportionate segments of the population who have been incarcerated or subject to a biased criminal justice system[121,122] and 2) new challenges posed to voting related to access (e.g., time off of work, discrimination-based distrust of the system).[121,123] Poor health and lower voting rates have been associated in many studies without a clear explanation of which is a preceding factor. Individuals with poorer health have often a worse sense of political efficacy, concerns regarding voting accessibility (including in time), and competing social and financial burdens.[124] Notably, this data was only available for 2012 and then recollected in 2020 in Durham County; these 2012 data may introduce bias due to over and under reporting of election participation in certain communities in which population demographics shifted voting participation. However, to our knowledge, this is one of the only available data sets which has allowed linkage of local voter participation data in the primary election with health outcomes.

EDUCATION

Percent with bachelor's degree (American Community Survey)

The total number of residents aged 25 years or older who attained a bachelor's degree was ascertained in 2017 and 2018. Racial and ethnic disparities in educational opportunities have increased because of segregation, and racial inequities have been exacerbated by interpersonal bias and the inequitable distribution of resources to schools based in residential racial segregation and associated distribution of tax bases/wealth.[125] Despite interventions, disparities in what has been called "the most consequential attainment outcome – earning a postsecondary degree" have remained stagnant, reflecting the persistence of structural racism.[125] Lower educational attainment has been associated with chronic diseases including CKD, DM, and HTN due to lower health care access, less access to health-promoting interventions, and wealth.

Childcare Centers (Durham Compass)

The measure of childcare centers and family child-care homes licensed by the state of North Carolina's Division of Child Care and Early development from 2017-2018 per square mile (which does not include unlicensed locations). Access to child-care in early-child development has significant implications for families and long-term health outcomes. Structural racism impacts the availability and location of high-quality child-care centers, and studies have demonstrated that Black and Latino/a/e/Hispanic children are more likely to experience lower quality childcare. However, data regarding the quality of these centers is unavailable in our available data. Out of home childcare has been associated with greater family-level social and economic disadvantage[126], and other studies have suggested racially/ethnically minoritized Black and Latino/Hispanic children are more likely to be in Head Start versus White counterparts, who may have access to other early education opportunities.[127] In addition, child care center quality has been described as lower in more socially disadvantaged neighborhoods.[128] Finally, neighborhoods with greater spatial density of child care centers per square mile have been associated with higher rates of early child maltreatment referrals, suggesting need for greater contextualization of the settings in which high densities of lower quality centers occur.[129]

UNEMPLOYMENT

Percent unemployed (American Community Survey)

Unemployment was ascertained by determining the total number of residents aged 16 years or older in the civilian labor force but who were unemployed in 2017 and 2018 as a proportion of the total number of residents aged 16 years or older in the civilian labor force. Unemployment may reflect discriminatory hiring practices, which have not equitably distributed opportunity between minoritized vs. White individuals, which impacts health insurance and other critical

resource access.[130,131] Unemployment has also been associated with poor health outcomes, including CKD, DM, and HTN.

HEALTH CARE INSURANCE COVERAGE

Percent uninsured (American Community Survey)

Uninsurance was ascertained by determining the total number of individuals without any health insurance in 2017 and 2018, as a proportion of total adults. Lack of insurance may reflect a manifestation of structurally inequitable policies which have eliminated opportunities for non-employer-based health insurance and other mechanisms that provide universal coverage (e.g., Medicaid expansion).[132,133] Uninsurance has been associated with higher prevalence of CKD, DM, and HTN and poorer outcomes among individuals with a range of chronic health conditions.

COMPOSITE MEASURES OF STRUCTURAL RACISM

Percent Whiteness (American Community Survey)

The percent of the White population was ascertained for each census block group using data from 2017-2018. Block groups with a higher percentage of individuals racialized as White receive structural advantages including resources and health-impacting opportunities. This greater accrual of advantaging resources (versus neighborhoods with a lower % of the population racialized as White) is associated with lower chronic disease prevalence.

Area deprivation index (ADI)

ADI was ascertained in 2015 through linkage of neighborhoods to the publicly available ADI state rankings in deciles[62], in which a higher decile corresponds to more deprivation. The area deprivation index integrates key values from 18 Census variables including poverty, education, housing, and unemployment to characterize the level of deprivation in a certain region, and has been used to aid with targeted resource allocation and associated with numerous health outcomes including all-cause mortality, cervical cancer, and Alzheimer's disease.[62]

Index of Concentrations at the Extremes (ICE) (race-income)

ICE race-income was calculated using data from 2016-2017. ICE is the ratio of the difference between extremely "advantaged" and extremely "disadvantaged" units (e.g., individuals or households) to the total number of units in the region of interest, where the definitions of "advantaged" and "disadvantaged" depend on the construct of interest. For example, if the measure is income, then those in the 80th percentile and 20th percentile might represent advantaged and disadvantaged groups, respectively. Similarly, if the measure is race, White and Black might be the comparison groups. Following the methodology developed by Krieger et al, the combination ICE for race and income used in this study compared households with a White householder and income ≥$100,000 to those with a Black householder and income <$20,000. The ICE-RI was calculated at the census tract level due to income data by householder race being unavailable at the block group level. This may limit the accuracy of the extrapolation of this data to the BG level. We further identified structural racism via the spatial and racial/economic polarization of communities across neighborhoods due to redlining and other structurally racist policies, which created and sustained spatial polarization of neighborhoods. Racialized economic segregation operationalized by ICE-race income measures have been associated with a range of negative health outcomes in multiple studies and has been used a proxy for structurally racist policies that concentrated wealth/advantage in primarily White neighborhoods.[46,59]

Race, racism, and structural disadvantage

A fundamental tenet these analyses held is that race is the primary sociopolitical – not biological – modifier through which structural racism relates to affect health.[76-85] Because structural racism has a differential effect on individuals according to their racialization as White or as a minoritized person (i.e., less effects on White individuals versus racially minoritized individuals),[50-54] we sought to describe various socio-structural features of each neighborhood as categorized by the

© 2023 Mohottige D et al. *JAMA Network Open.*

% of White individuals of each neighborhood.[53,55] We also examined the association between measures that discretely describe manifestations of structural racism and ICE-race-income on health outcomes. For instance, the Index of Concentration at the Extremes has been used to measure spatial polarization of deprived and socially privileged groups (by race and income), and has been used as a proxy for structural racism in association with pre-term birth, infant mortality[8,9,59] While there are nuances in the experiences of individuals racialized as White and as racial and ethnic minorities across race and ethnicity, the accumulation of privilege over generations/time is a unique feature for individuals racialized as White, as is the aggregation of these resources in areas in which a greater percentage of White individuals reside.[134]

© 2023 Mohottige D et al. *JAMA Network Open.*

eAppendix 2. Structural racism variables, direction associated with more structural racism, and association with structural racism

**Variable**	**Direction associated with more SR**	**Referenced evidence of relation to SR**
% White Population	Decreased	Areas with lower % White population experience greater structural racism/disadvantage (historically due to redlining and present-day disinvestment)
Ice Race Income White ≥ $100k	Decreased	Areas with lower % of high-income White individuals experience greater structural disadvantage due to disinvestment and less access to structural advantages
Area deprivation index (ADI)	Increased	Areas with greater overall disadvantage experience more manifestations of structural racism including structural disadvantages and greater disinvestment
Child care centers	Increased	More child care centers exist in communities with higher % of racial/ethnic minority individuals
Homes near bus stops	Increased	More public transit stops exist in communities with a higher % of racial/ethnic minority individuals
Tree cover	Decreased	Less tree cover exists in communities with a higher % of racial/ethnic minority individuals due to structurally racist environmental policies and opportunity
Violent crime reporting	Increased	More criminalization/policing occurs in communities that have a higher % of racial/ethnic minority individuals

© 2023 Mohottige D et al. *JAMA Network Open.*

Impervious area	Increased	More impervious areas (where water cannot free flow) occurs in communities with a higher % of racial/ethnic minority individuals
Eviction rate	Increased	More evictions occur in communities with a higher % of racial/ethnic minority individuals
Primary election participation	Decreased	Less voting participation occurs in communities with a higher % of racial/ethnic minority individuals due to gerrymandering and other policies
Median household income ($)	Decreased	Lower median household incomes characterize communities with a higher % of racial/ethnic minority individuals due to racial wage inequality and job discrimination
Poverty rate	Increased	Higher poverty occurs in communities with a higher % of racial/ethnic minority individuals due to the lack of poverty reducing policies/structural interventions
% Bachelor’s degree	Decreased	Lower% completion of bachelor’s degree training occurs in communities with a higher % of racial/ethnic minority individuals due to racial inequalities in educational opportunity
% Unemployed	Increased	Higher unemployment occurs in areas with higher % of racial/ethnic minority individuals due lack of structural interventions to ensure job opportunity and equal hiring
% Uninsured	Increased	Higher % uninsurance occurs in areas with

© 2023 Mohottige D et al. *JAMA Network Open.*

		higher % of racial/ethnic minority individuals due to the lack of policies to ensure insurance equality (e.g., Medicaid expansion)
Police shootings	Increased	Structural racism has been associated with more criminalization and policing

© 2023 Mohottige D et al. *JAMA Network Open.*

eReferences.

96. Inker LA, Astor BC, Fox CH, et al. KDOQI US commentary on the 2012 KDIGO clinical practice guideline for the evaluation and management of CKD. Am J Kidney Dis. 2014;63(5):713-735. Medline:24647050 doi:10.1053/j.ajkd.2014.01.416
97. Inker LA, Schmid CH, Tighiouart H, et al; CKD-EPI Investigators. Estimating glomerular filtration rate from serum creatinine and cystatin C. N Engl J Med. 2012;367(1):20-29. Medline:22762315 doi:10.1056/NEJMoa1114248
98. Delgado C, Baweja M, Crews DC, et al. A unifying approach for GFR estimation: recommendations of the NKF-ASN Task Force on Reassessing the Inclusion of Race in Diagnosing Kidney Disease. J Am Soc Nephrol. 2021;32(12):2994-3015. Medline:34556489 doi:10.1681/ASN.2021070988
99. Eneanya ND, Boulware LE, Tsai J, et al. Health inequities and the inappropriate use of race in nephrology. Nat Rev Nephrol. 2022;18(2):84-94. Medline:34750551 doi:10.1038/s41581-021-00501-8
100. Norris KC, Eneanya ND, Boulware LE. Removal of race from estimates of kidney function: first, do no harm. JAMA. 2021;325(2):135-137. Medline:33263722
101. Brabec E, Schulte S, Richards PL. Impervious surfaces and water quality: a review of current literature and its implications for watershed planning. J Plann Lit. 2002;16(4):499-514. doi:10.1177/088541202400903563
102. Syed ST, Gerber BS, Sharp LK. Traveling towards disease: transportation barriers to health care access. J Community Health. 2013;38(5):976-993. Medline:23543372 doi:10.1007/s10900-013-9681-1
103. Ulmer JM, Wolf KL, Backman DR, et al. Multiple health benefits of urban tree canopy: The mounting evidence for a green prescription. Health Place. 2016;42:54-62. Medline:27639106 doi:10.1016/j.healthplace.2016.08.011
104. Merkin SS, Coresh J, Diez Roux AV, Taylor HA, Powe NR. Area socioeconomic status and progressive CKD: the Atherosclerosis Risk in Communities (ARIC) study. Am J Kidney Dis. 2005;46(2):203-213. Medline:16112038 doi:10.1053/j.ajkd.2005.04.033
105. Krop JS, Coresh J, Chambless LE, et al. A community-based study of explanatory factors for the excess risk for early renal function decline in blacks vs whites with diabetes: the Atherosclerosis Risk in Communities study. Arch Intern Med. 1999;159(15):1777-1783. Medline:10448782 doi:10.1001/archinte.159.15.1777
106. Crews DC, Novick TK. Social determinants of CKD hotspots. Semin Nephrol. 2019;39(3):256-262. Medline:31054624 doi:10.1016/j.semnephrol.2019.02.003
107. Nicholas SB, Kalantar-Zadeh K, Norris KC. Socioeconomic disparities in chronic kidney disease. Adv Chronic Kidney Dis. 2015;22(1):6-15. Medline:25573507 doi:10.1053/j.ackd.2014.07.002
108. Desmond M, Gershenson C. Who gets evicted: assessing individual, neighborhood, and network factors. Soc Sci Res. 2017;62:362-377. Medline:28126112 doi:10.1016/j.ssresearch.2016.08.017
109. Greenberg D, Gershenson C, Desmond M. Discrimination in evictions: empirical evidence and legal challenges. Harv CR-CLL Rev. 2016;51:115.
110. Mah J. Gentrification-induced displacement in Detroit, Michigan: An analysis of evictions. Hous Policy Debate. 2021;31(3-5):446-468. doi:10.1080/10511482.2020.1800781
111. Iguchi MY, Bell J, Ramchand RN, Fain T. How criminal system racial disparities may translate into health disparities. J Health Care Poor Underserved. 2005;16(4)(suppl B):48-56. Medline:16327107 doi:10.1353/hpu.2005.0081
112. Jackson DB, Posick C, Vaughn MG. New evidence of the nexus between neighborhood violence, perceptions of danger, and child health. Health Aff (Millwood). 2019;38(5):746-754. Medline:31059369 doi:10.1377/hlthaff.2018.05127

© 2023 Mohottige D et al. *JAMA Network Open.*

113. Theall KP, Shirtcliff EA, Dismukes AR, Wallace M, Drury SS. Association between neighborhood violence and biological stress in children. JAMA Pediatr. 2017;171(1):53-60. Medline:27842189 doi:10.1001/jamapediatrics.2016.2321
114. Gilbert KL, Ray R. Why police kill black males with impunity: applying public health critical race praxis (PHCRP) to address the determinants of policing behaviors and “justifiable” homicides in the USA. J Urban Health. 2016;93(Suppl 1)(suppl 1):122-140. Medline:26661386 doi:10.1007/s11524-015-0005-x
115. Alang S, McAlpine D, McCreedy E, Hardeman R. Police brutality and Black health: setting the agenda for public health scholars. Am J Public Health. 2017;107(5):662-665. Medline:28323470 doi:10.2105/AJPH.2017.303691
116. Bor J, Venkataramani AS, Williams DR, Tsai AC. Police killings and their spillover effects on the mental health of Black Americans: a population-based, quasi-experimental study. Lancet. 2018;392(10144):302-310. Medline:29937193 doi:10.1016/S0140-6736(18)31130-9
117. Cooper HL, Fullilove M. Excessive police violence as a public health issue. J Urban Health. 2016;93(Suppl 1)(suppl 1):1-7. Medline:26984303 doi:10.1007/s11524-016-0040-2
118. Bailey ZD, Feldman JM, Bassett MT. How structural racism works—racist policies as a root cause of U.S. racial health inequities. N Engl J Med. 2021;384(8):768-773. Medline:33326717 doi:10.1056/NEJMms2025396
119. Edwards F, Lee H, Esposito M. Risk of being killed by police use of force in the United States by age, race-ethnicity, and sex. Proc Natl Acad Sci U S A. 2019;116(34):16793-16798. Medline:31383756 doi:10.1073/pnas.1821204116
120. Blakely TA, Kennedy BP, Kawachi I. Socioeconomic inequality in voting participation and self-rated health. Am J Public Health. 2001;91(1):99-104. Medline:11189832 doi:10.2105/AJPH.91.1.99
121. Uggen C, Manza J. Democratic contraction: political consequences of felon disenfranchisement in the United States. Am Sociol Rev. 2002;67(6):777-803. doi:10.1177/000312240206700601
122. Hjalmarsson R, Lopez M. Erratum to “The voting behavior of young disenfranchised felons: would they vote if they could?”. Am Law Econ Rev. 2017;19(2):504. doi:10.1093/aler/ahx018
123. Epperly B, Witko C, Strickler R, White P. Rule by violence, rule by law: lynching, Jim Crow, and the continuing evolution of voter suppression in the U.S. Perspect Polit. 2020;18(3):756-769. doi:10.1017/S1537592718003584
124. Brown CL, Raza D, Pinto AD. Voting, health and interventions in healthcare settings: a scoping review. Public Health Rev. 2020;41:16-16. Medline:32626605 doi:10.1186/s40985-020-00133-6
125. Merolla DM, Jackson O. Structural racism as the fundamental cause of the academic achievement gap. Sociol Compass. 2019;13(6):e12696. doi:10.1111/soc4.12696
<eref>126. US Department of the Treasury. The economics of child care supply in the United States. Accessed November 15, 2023. https://home.treasury.gov/system/files/136/The-Economics-of-Childcare-Supply-09-14-final.pdf</eref>
127. Hillemeier MM, Morgan PL, Farkas G, Maczuga SA. Quality disparities in child care for at-risk children: comparing Head Start and non–Head Start settings. Matern Child Health J. 2013;17(1):180-188. Medline:22392601 doi:10.1007/s10995-012-0961-7
128. Burchinal M, Nelson L, Carlson M, Brooks-Gunn J. Neighborhood Characteristics, and Child Care Type and Quality. Early Educ Dev. 2008;19:702-725. doi:10.1080/10409280802375273
129. Klein S. The availability of neighborhood early care and education resources and the maltreatment of young children. Child Maltreat. 2011;16(4):300-311. Medline:22114183 doi:10.1177/1077559511428801
130. Darity WA Jr. Employment discrimination, segregation, and health. Am J Public Health. 2003;93(2):226-231. Medline:12554574 doi:10.2105/AJPH.93.2.226

© 2023 Mohottige D et al. *JAMA Network Open.*

131. Stewart LD, Perlow R. Applicant race, job status, and racial attitude as predictors of employment discrimination. J Bus Psychol. 2001;16(2):259-275. doi:10.1023/A:1011113301301
132. Buchmueller TC, Levy HG. The ACA's impact on racial and ethnic disparities in health insurance coverage and access to care. Health Aff (Millwood). 2020;39(3):395-402. Medline:32119625 doi:10.1377/hlthaff.2019.01394
133. Sohn H. Racial and ethnic disparities in health insurance coverage: dynamics of gaining and losing coverage over the life-course. Popul Res Policy Rev. 2017;36(2):181-201. Medline:28366968 doi:10.1007/s11113-016-9416-y
134. Blacksher E, Valles SA. White privilege, White poverty: reckoning with class and race in America. Hastings Cent Rep. 2021;51(S1)(suppl 1):S51-S57. Medline:33630341 doi:10.1002/hast.1230

© 2023 Mohottige D et al. *JAMA Network Open.*
