# Supplementary material for: Residential Structural Racism and Prevalence of Chronic Health Conditions
Source: JAMA Netw Open. 2023 Dec 21;6(12):e2348914. doi: 10.1001/jamanetworkopen.2023.48914 (PMC10739116; doi:10.1001/jamanetworkopen.2023.48914)
Supplement: Supplement 2. — Data Sharing Statement [file jamanetwopen-e2348914-s002.pdf]

Data Sharing Statement

Mohottige. Residential Structural Racism and Prevalence of Chronic Health Conditions. *JAMA Netw Open*. Published December 21, 2023. doi:10.1001/jamanetworkopen.2023.48914

Data
Data available: Yes
Data types: Deidentified participant data
How to access data: https://compass.durhamnc.gov/en/
When available: With publication

Supporting Documents
Document types: None

Additional Information
Who can access the data: https://compass.durhamnc.gov/en/
Types of analyses: code can be made available after pub w/request
Mechanisms of data availability: code can be made available after pub w/request
Any additional restrictions: NA
